# Who's connected to whom and how: a model of evolving relationships and roles in faculty development and curriculum development during curriculum renewal and innovation

**DOI:** 10.15694/mep.2018.0000119.1

**Published:** 2018-06-07

**Authors:** Jana Lazor, Susan Glover Takahashi, Karen Leslie

**Affiliations:** 1 Faculty of Medicine

**Keywords:** Faculty Development, Curriculum Renewal and Innovation, Educator Development, Teacher Development

## Abstract

This article was migrated. The article was marked as recommended.

Faculty development and curriculum development are essential to the work of academic health sciences institutions. Through collegial conversations, more intense dialogue, and ‘workshopping’, we have identified a new model of how these two practices can be effectively integrated. We propose that this new model can create a system of knowledge mobilization and quality improvement that will greatly enhance curricular renewal and innovation. We invite and welcome comments and feedback from the health professions education community.

## Introduction

Health care education should prepare health professionals for the rapidly changing needs of patients’ and society. Concurrently, educators are responsible to ensure the curriculum for health professionals continues to evolve through major renewals and meaningful innovation and revision ^1^. Successful curricular renewal needs to incorporate evidence-informed education while staying open to innovation and discovery with a focus on continuous organizational learning. Additionally, educators creating curriculum, front line teachers implementing and delivering the curriculum, and organizations sponsoring these curricular changes, all need to be appropriately supported. A key enabler of curriculum change is faculty development practice that supports educators, teachers, leaders and the organization.

As the authors, we found ourselves in different contexts as faculty developers responsible for developing, implementing and evaluating various faculty development strategies during major curricular changes in the undergraduate (MD Program) ^1^ and post graduate (post MD Program) ^2^ programs in the Faculty of Medicine at the University of Toronto. Initially, these roles were relatively independent from each other, however more recently university leaders created an opportunity to integrate structurally the faculty development leads for the MD Program and post MD Program with the Faculty of Medicine’s Centre of Faculty Development.

**We asked the question:** *What should be the relationship and connection between the practices of faculty development and curriculum development during a time of major curriculum renewal and innovation?*

We reflected collaboratively on our various experiences to examine this topic critically and tested our ideas through workshops at national and international conferences. This resulted in and generated the preliminary components of a new framework for the practice of faculty development during curricular change to meet the rapidly evolving changes in health professional education programs.

The purpose of this article is to:

• Share the developed framework for the practice of faculty development and its relationship to the practice of curriculum work during major curriculum change.
• Encourage dialogue about models for the practice of faculty development and practice of curriculum development during times of curriculum change.

## Background

Faculty development can be described as an organizational strategy to support faculty in their academic roles as educators, teachers, leaders and scholars. Faculty development has been clearly identified as a key enabler of change in a health professions curriculum. ^3- 5^ A number of authors have written about faculty development “for” curriculum change, ^6- 8^ as a key driver for change. At the same time, many have identified a gap in the literature regarding what this practice might look like and how faculty development could be effectively integrated into curriculum work. ^7^ There is considerable value in ongoing and reciprocal relationships between curriculum work and faculty development when change is required. ^6^ This is particularly true when there is rapid curriculum change, so that the required faculty development processes and resources are ready when the curriculum is ready. Thus, the concurrent development of faculty development with the curriculum design development improves quality and also ‘saves time’. Onyura et al discovered that best evidence from health professions education research was not routinely implemented in the curriculum due to a number of barriers but faculty development was identified as an enabler in this process. ^9^ Re-conceptualizing faculty development during times of curriculum change as knowledge mobilization, rather than just teacher preparation, may be a critical new lens with which the practice may need to be redefined. ^10^

In the literature we see faculty development positioned in different ways during curriculum change as either: key to implementation; ^8^ driving the need for change; ^6^ getting the teachers ready to teach differently; ^11^ or preparing faculty to assess differently. ^12^ What is missing is an integrated and holistic approach to align key educational practices during curriculum change.

After reviewing the existing literature, we found that the practice of faculty development was not articulated in a way that could help faculty development and curriculum practitioners understand how to best align their two education practices. This is essential to optimize collaboration and productivity and minimize barriers.

**Figure 1.**
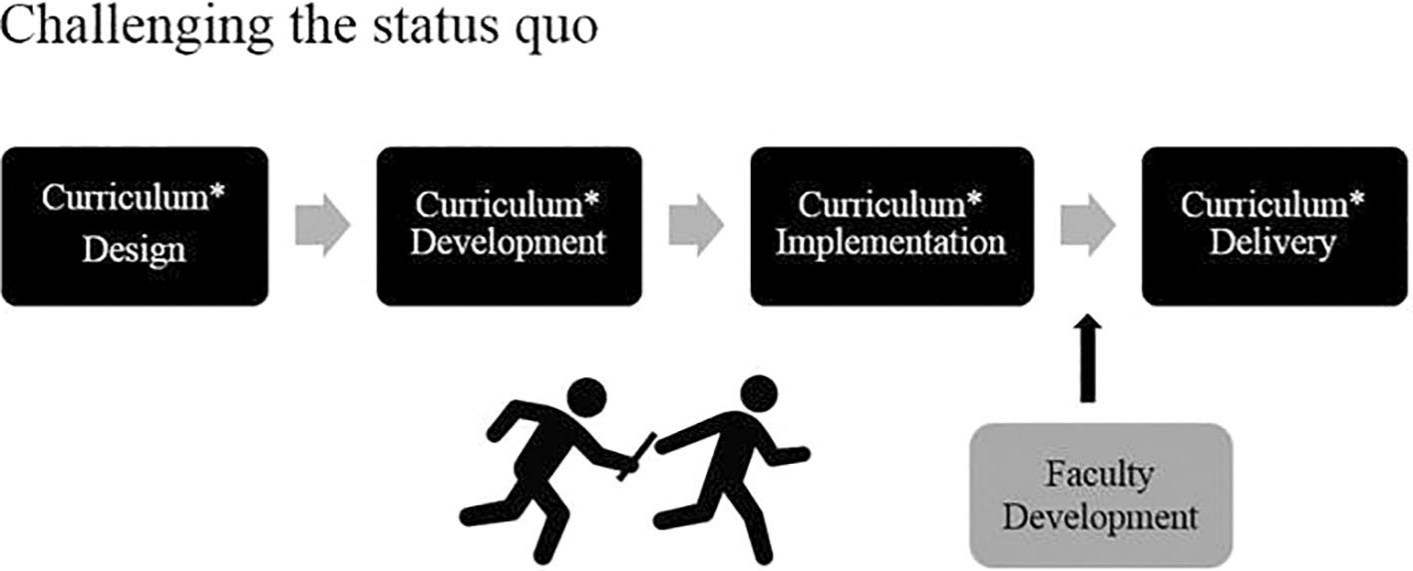
Relay model of faculty development and curriculum work.

We aim to challenge the *status quo* that often positions faculty development as the anchor in a ‘relay’-type model where curriculum design, development and implementation precede and then ‘hand off’ to faculty development for final delivery (see Figure 1). Faculty who are responsible for designing and developing the curriculum may benefit from faculty development support to assist with the transfer of education theory into practice and to provide a sound approach to the design and development. ^7^ The design and development of the curriculum has direct and significant implications for the faculty who will be asked to implement and deliver the front-line teaching and there is a need for a voice that faculty development can provide to advocate for faculty needs and abilities to operationalize the curricular vision effectively. Waiting until the curriculum is designed, developed and finalized before involving faculty development may not provide adequate time to design, develop and implement an effective faculty development strategy, which ultimately impacts the desired and intended outcomes of the curriculum.

When timelines are short (as they often are), there may be insufficient time before curricular delivery for faculty developers to create the resources, programming and supports required to prepare faculty teachers adequately and there is rarely the ‘room’ to make changes to the curricular expectations if any questions arise from the faculty developers or teachers.

**Figure 2.**
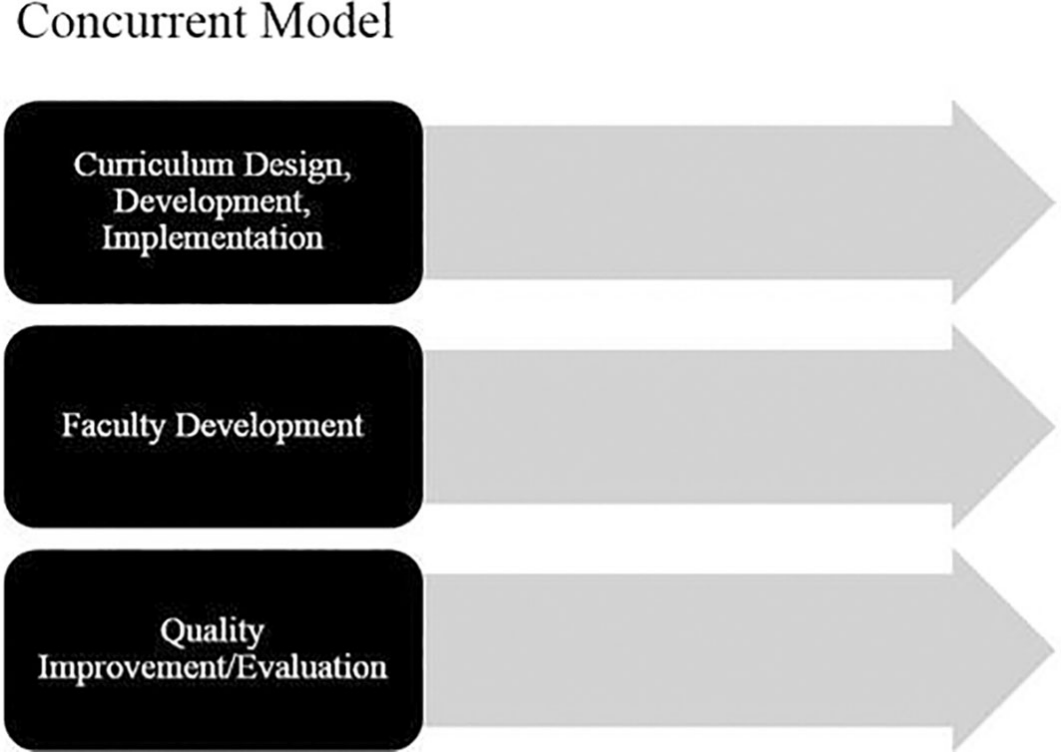
The concurrent model of faculty development and curriculum work.

Some may suggest that the solution to these limitations is the concurrent model where curriculum work proceeds in parallel with faculty development and quality improvement/evaluation planning (see Figure 2). This alternative approach may provide more time for development of faculty resources and learning activities, however there is risk for the three pathways to develop in a misaligned fashion if there is not adequate dialogue and shared understanding of the vision and expectations. This misalignment for learners and teachers can result in disengagement for both. ^13^

We strongly believe there is both a need and an opportunity for a new framework or model of practice that will *align and integrate* curriculum and faculty development educational practices that are essential to effective curriculum renewal and innovation.

## Process of the model development to date

We used a seven step process to develop the model to date. Each step will be described.

• **STEP 1:** Identification of the limitations of the current way in which we are conceptualizing the practice of faculty development during curriculum renewal and innovation
• **STEP 2:** Critical reflection on the nature of the relationship between faculty development and curriculum development during curriculum renewal and innovation
• **STEP 3**: Description of curriculum work components
• **STEP 4:** Consideration of where faculty development needs to be located within the curriculum work
• **STEP 5:** Identification of key roles, tasks and goals required to support a health professions education system undergoing curriculum change
• **STEP 6:** Gathering of input from the local, national, and international faculty development community through presentations of workshops at the 4 ^th^ International Conference of Faculty Development (2017) and the Canadian Conference on Medical Education (2018). We asked the participants to illustrate how the process works at their schools and then, after discussion of our cases, to think and revise their models. Appendix 1 outlines the activities participants engaged in and some images of initial models and their development throughout the workshop.
• **STEP 7:** Using the experience and feedback from the workshops to revise the model.

## Description of a new model

### Components

The models starts by defining five distinct components involved in curriculum work. ^6^ When we refer to the term “curriculum” we mean the objectives or outcomes; the teaching and learning content; the teaching paradigms and learning science concepts used; and learner assessment. These include curriculum:

1. Design
2. Development
3. Implementation
4. Delivery
5. Evaluation

**Figure 3.**
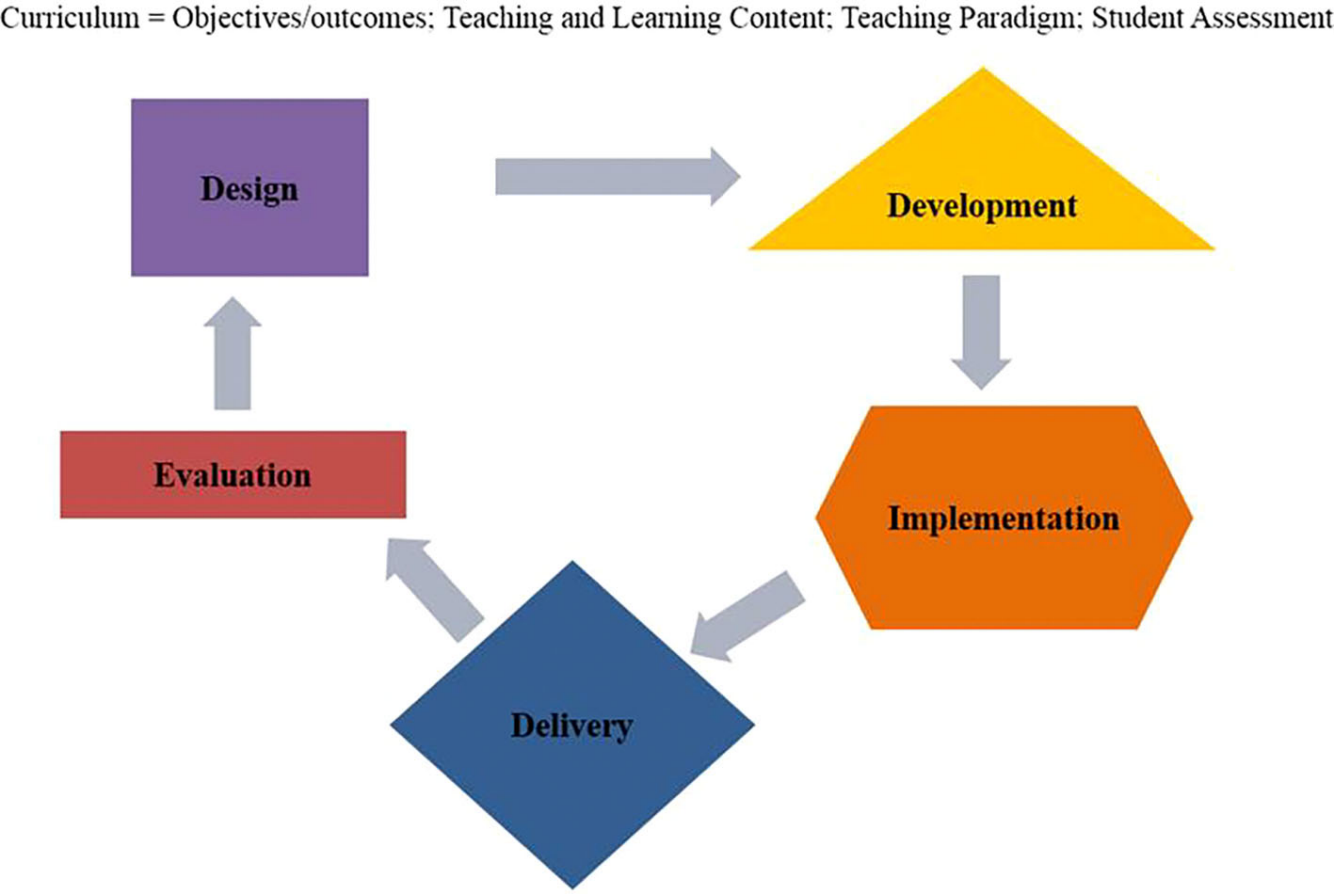
Curriculum Work.

These five components are connected to each other and the process is cyclical, interdependent and iterative in nature, rather than linear and static (see Figure 3). See appendix 2 for descriptions of each component.

A key component of curriculum work is also faculty development. We asked ourselves: “Where does faculty development fit into this structure? How should we conceptualize the practice of faculty development?” When considering where the practice of faculty development fits, we need to challenge the assumption often held in health care education, that faculty developers belong primarily in the curriculum implementation and delivery stages. We propose that practice of faculty development should be integrated into each of these five stages - which we call “tables of work”. Therefore, when the question is asked “Who needs to be at your table?” for identifying committees, working groups, and assigning responsibilities; the answer needs to be inclusive of faculty who consciously and strategically bring the lens of faculty development.

**Figure 4.**
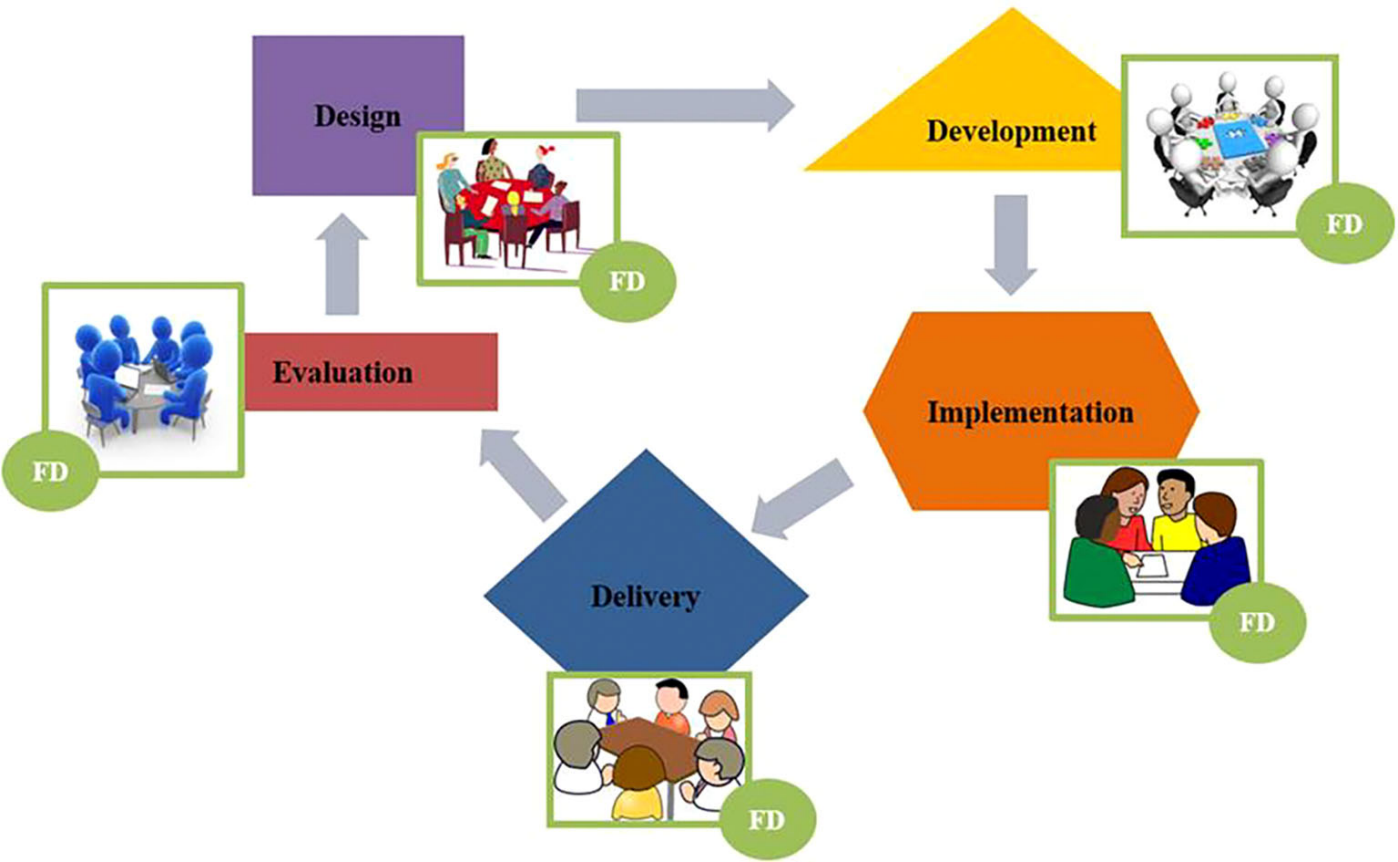
Integrated model of faculty development and curriculum work. FD = Faculty Development

## Faculty development roles and responsibilities in a new model

**Figure 5.**
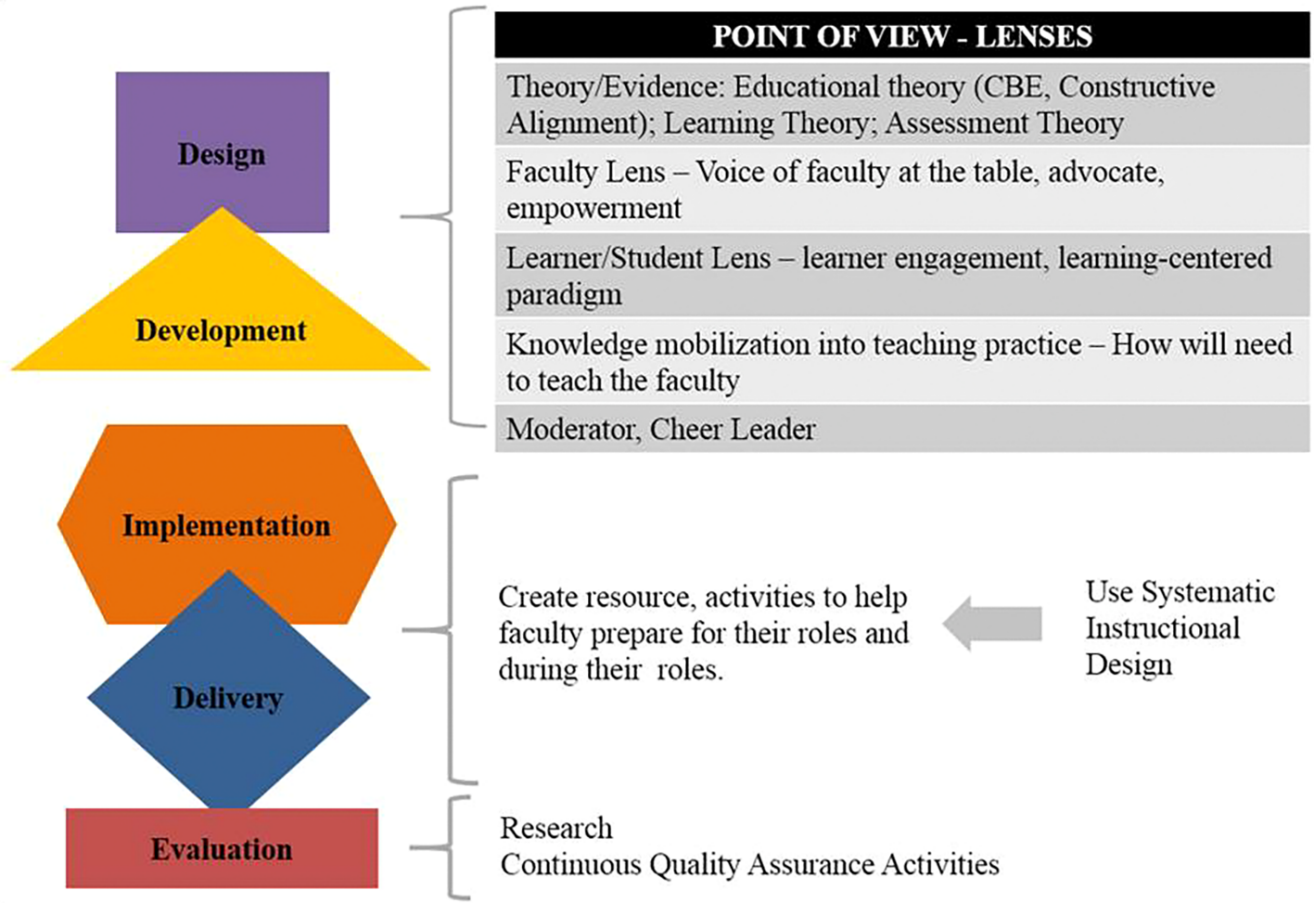
Point of view - lenses.

Following is a list of potential faculty development activities and roles at different stages of curriculum work as illustrated in Figure 5.

### Curriculum Design and Development

During the curriculum design and development stages, a faculty development practitioner could bring into conversations and activities different points of view including:

• Theory and evidence: educational and learning theory; evidence informed practice; assessment theories or best practices
• Faculty member lens: voice of faculty at the table, advocacy and faculty empowerment
• Learner or student lens: learner engagement, learning-centered paradigm
• Knowledge mobilization into teaching practices: consideration of the impact of change on faculty and how this can be incorporated into their teaching practice
• Facilitator, moderator, cheer leader.

### Curriculum Implementation and Delivery

During curriculum implementation and delivery, faculty development needs to focus on the creation of resources and activities to help faculty during preparation and their teaching roles. This also needs to be done in collaboration with the curriculum developers and aligned with the intended design and expectations. At this stage it is useful to identify specific teaching roles, for example Case-Based Learning tutor vs lecturer vs clinical skills tutor vs clinical preceptor. For each specific role, a thorough assessment is needed to determine the following:

• Who are the teachers who will be or have been assigned to these roles?
• What will be their specific tasks relating to teaching and student assessment (task analysis)?
• For the individual faculty member, what is different from before and what is the same?
• Where are the faculty located and where will they be teaching?
• How to make the resources and activities accessible? The use of a structured instructional design process is essential. ^14^

### Curriculum Evaluation

The evaluation of the curriculum on a programmatic level offers an opportunity for scholarly work within the practice of faculty development and also for continuous quality assurance activities. Both of these can generate data about the effectiveness of the faculty development strategy and also provide insights into the different curriculum components such as:

• the actual role and experience of the teacher
• learner engagement
• teacher engagement
• the need for addition targeted faculty development (resources or activities)
• the status of alignment between curriculum, assessment, and faculty development.

## Goals of faculty development in the new model

The practice of faculty development, during curriculum renewal and innovation, has not been as well described as curriculum development practice. We suggest that in the proposed model of how these two practices can align and integrate, the primary focus for the practice of faculty development in this context is to support faculty in their academic roles as educators and teachers.

Through this model, we propose that the goals for the practice of faculty development during curriculum change should extend to include:

• Knowledge mobilization, ^10^
• Knowledge creation or knowledge building, ^15^
• Continuous quality Improvement (faculty development as improvement science), ^16^
• Supporting the change process and managing transitions, ^17, 18^
• Fostering organizational learning. ^19^

## Conclusions

We propose an education practice model during curriculum renewal and innovation that integrates and aligns curriculum practice and faculty development practice across the entire curriculum process. There are limitations when incorporating faculty development at the end of the line or even when it is implemented in parallel with curriculum work. What is needed is an integrated and aligned approach that can be rapidly responsive, with one practice influencing and supporting the other. The practice of faculty development within the integrated model can take on a variety of different roles such as: knowledge mobilization; knowledge creation; continuous quality improvement; and organizational learning. Future work for this model will involve describing the specific tasks or functions and strategies for faculty, at each of the curriculum steps, from the perspective of faculty responsible for the curriculum and faculty responsible for faculty development. An essential area of future development should include management of these evolving relations and roles in faculty development and curriculum development during different contexts during curriculum renewal and innovation.

We welcome feedback from the health care education community.

## Take Home Messages

• The integration of faculty development and curriculum development practices allows for a shared mental model that can improve overall effectiveness and efficiency of the curriculum renewal process
• Think of faculty development at the start and throughout all the stages of curriculum renewal and innovation.

## Notes On Contributors

Dr. Jana Lazor is the Director of Faculty Development, Office of Faculty Development, MD Program, a Senior Integrated Scholar MD Education/CFD with the Centre for Faculty Development, and an Associate Professor, Department of Community Medicine, Faculty of Medicine, University of Toronto.

Dr. Susan Glover Takahashi is the Director of Education, Innovation and Research, Post Graduate Medical Education, a Senior Integrated Scholar PostMD/CFD with the Centre for Faculty Development, and an Associate Professor, Department of Community Medicine, Faculty of Medicine, University of Toronto.

Dr. Karen Leslie is the Director of the Center for Faculty Development and a Clinician Educator and Professor of Paediatrics in the Department of Paediatrics, Faculty of Medicine, University of Toronto.

## Appendices

### Appendix 1. Samples from the workshops and attendees activities that helped to refine the model

**Activity:** Models of faculty development in relation to curriculum development:

**Question:** Where do you position FD at your school?

**Task:** Using the shapes that represent the various stages of curriculum design, development, implementation and evaluation, as well as faculty development involvement, create a picture of where most of your table’s participants position FD.

**Shapes that participants were provided to work with:**

**Example of initial frameworks:**

**Example of how participants modified the frameworks after they had a chance to engage in discussion of our two case studies:**

**MD Program Foundations Curriculum Renewal and Post MD Program, implementation of Competency Based Education.**

### Appendix 2. Working Definitions of Key Components

**Table:**

#	Key Components	Description
1	Curriculum Design	**Curriculum design** refers to the organizing framework, principles, philosophical or educational approaches, goals or outcomes, and/or key features for the curricular innovation. For example, a competency based innovation may include developmental stages, entrustable professional activities and workplace based assessments. Or the curriculum may be informed by specific theories such as adaptive expertise, learning for understanding, discovery learning, contextual variation, and/or programmatic assessment.
2	Curriculum Development	**Curriculum development** refers to the planning and content details related to the curricular innovation. Represents the teaching and learning materials and assessments. For example, a competency based innovation may decide on 4 developmental stages, develop the inventory of entrustable professional activities (EPAs) and outline the criteria and scales that will be used in workplace based assessments. In an integrated case-based curriculum it could represent the case, tutor guides, lectures, learning resources, and construction of test items.
3	Curriculum Delivery	**Curriculum delivery** refers to how learners, teachers, content and context for the curriculum innovation are experienced or enacted. It is the front line teaching and student assessment. For example, a competency based innovation may use an online platform to record and monitor the developmental stages, entrustable professional activities and workplace based assessments. The content may be taught in seminars and in the clinical setting by a wide range of health professionals or in small group case-based tutorials by Case-Based Learning (CBL) tutors. Individuals who deliver the curriculum may or may not be the same people who developed the curriculum materials.
4	Curriculum Implementation	**Curriculum implementation** refers to project management elements in preparation to the launch and ongoing operations of the curricular innovation. For example, a competency based innovation may have a 3 to 6 to 12-month planning process. Implementation may involve teacher recruitment, scheduling, distribution of teaching materials, triggering when it is time to complete as assessment. A new CBL integrated curriculum may involve faculty knowing how to access their virtual cases and tutor guides on line.
5	Curriculum Evaluation	**Curriculum evaluation** refers to program evaluation that monitors what aspects of the curricular innovation are working well and those that need refinement, restructuring or rethinking.
