# Supplementary material for: Who's connected to whom and how: a model of evolving relationships and roles in faculty development and curriculum development during curriculum renewal and innovation
Source: MedEdPublish (2016). 2018 Jun 7;7:119. [Version 1] doi: 10.15694/mep.2018.0000119.1 (PMC10699385; doi:10.15694/mep.2018.0000119.1)
Supplement: Peer review history

# Reviewer report: Reviewer response for version 1

**DOI:** 10.21956/mep.19595.r29096

**Published:** 2019-03-15

**Authors:** Richard Hays

**Affiliations:** 1 James Cook University

This review has been migrated. The reviewer awarded 4 stars out of 5 As someone involved mostly with new programs or major changes to programs, I recognise the model proposed here and feel that I have taken the journey several times. It was my experience that aligning faculty development to a need for change is both sound and powerful, because it gets “buy in”. I would add that working with the learners/students is also important, as they are the recipients of the changed programs and learning strategies. Their views can improve development through new ideas and stronger engagement. This also helps with evaluation, as more participants (faculty and learners) have a stake when they have been part of the implementation, and so respond to evaluation methods. So, how well does this work in a larger, well established and stable institution where “normal business” dominates? The authors work in one of North America’s larger medical education groups, so how can the model be applied in other contexts? The definition of “faculty” may need a little discussion, as in North America there is usually a strong relationship between medical programs and dispersed clinical teachers. How can the model be implanted where the relationships are less direct? I recommend this paper to all involved in facul y devthe lopment, particularly when major curriculum change is contemplated.

Reviewer Expertise:

NA

No decision status is available

# Reviewer report: Reviewer response for version 1

**DOI:** 10.21956/mep.19595.r29095

**Published:** 2018-06-07

**Authors:** P Ravi Shankar

**Affiliations:** 1 American International Medical University

This review has been migrated. The reviewer awarded 4 stars out of 5 Curriculum renewal and innovation occupies an important place in medical education. The authors of this well-written paper put forward the view that faculty members should be involved in each stage of the process and not just at the implementation and delivery stages. The large number of faculty available at well-established medical schools may allow medical educators not to be directly involved in student teaching-learning. At most offshore Caribbean medical schools (OCMS) the number of faculty is small, faculty members wear many hats and most educators are actively involved in student learning. The Curriculum Committee serves as the primary body to bring together educators, teaching faculty and students to discuss curricular changes. I agree with the authors that involving teaching faculty in each stage of the process can enhance and strengthen curricular innovations. The figures add significantly to the understanding of the paper. I would be interested in knowing how the authors involved their faculty members during each stage of the curriculum renewal and innovation process and what exactly were the curricular changes implemented. This, however, may be the subject of another paper from the authors.

Reviewer Expertise:

NA

No decision status is available
